# Neuroanatomy education through the years: Revisiting the teaching slides used by Sir Wilfrid Edward Le Gros Clark

**DOI:** 10.1111/joa.70235

**Published:** 2026-09-08

**Authors:** Shiva A. Nischal, Kush M. Kale, Katherine Tourigny, Mohammed Abuelem, Gretchen Greene, Rachel Walsh, Zoltán Molnár

**Affiliations:** ^1^ St John's College University of Oxford Oxford UK; ^2^ Department of Physiology, Anatomy & Genetics University of Oxford Oxford UK; ^3^ Department of Clinical Neurosciences University of Oxford Oxford UK; ^4^ Division of Neurosurgery Dalhousie University Halifax Nova Scotia Canada; ^5^ National Institute of Mental Health, NIH Bethesda Maryland USA; ^6^ Feinberg School of Medicine Northwestern University Chicago Illinois USA; ^7^ National Institute of Neurological Disorders and Stroke, NIH Bethesda Maryland USA

**Keywords:** clinical medicine, history of medicine, medical education, Neuroanatomy, pedagogy

## Abstract

Sir Wilfrid Edward Le Gros Clark (1895–1971) was a pioneering British anatomist, physician, and evolutionary biologist whose legacy spans the foundational development of twentieth century neuroanatomy, primate biology, and medical education. From his formative experiences as a young medical officer in the First World War to his later academic ascendancy at the University of Oxford, Le Gros Clark exemplified a rare fusion of clinical insight, anatomical precision, and philosophical curiosity. His tenure in Borneo, where he studied primates in their natural habitats, enriched his evolutionary perspective, while his later role in debunking the Piltdown Man fraud cemented his place as a steadfast defender of scientific integrity. At Oxford, Le Gros Clark assembled a singular collection of neuroanatomical teaching specimens, many of which remain preserved today within the Department of Physiology, Anatomy & Genetics, University of Oxford. Through detailed analysis of selected slides, including cases of tabes dorsalis, disseminated sclerosis, amyotrophic lateral sclerosis, syringomyelia, and the pineal gland's *nervus conarii*, we reflect on how Le Gros Clark's anatomical interpretations anticipated many of the concepts central to contemporary neuroscience, and on how his legacy has influenced generations of students and scientists. What emerges from these materials is that Le Gros Clark taught students not simply to memorize, but to observe, interpret, and understand the functional significance of anatomical structures in health and disease.

## Graphical abstract

This paper examines the life and legacy of British anatomist Sir Wilfrid Le Gros Clark through his neuroanatomical teaching collection at the University of Oxford. The authors catalogued 103 specimens and analyzed selected slides—including cases of multiple sclerosis, amyotrophic lateral sclerosis, tabes dorsalis, syringomyelia, and the pineal gland's *nervus conarii*—alongside annotations and previously unpublished archival materials. The specimens show how Le Gros Clark connected visible anatomical lesions with patients' neurological symptoms, anticipating concepts that remain central to contemporary neuroscience. The paper argues that his enduring pedagogical contribution was teaching students to interpret anatomy through structure, function, and disease rather than simply memorizing it.

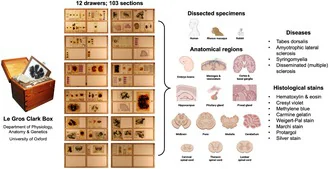

## 1 INTRODUCTION

Sir Wilfrid Edward Le Gros Clark was among the foremost anatomists and evolutionary biologists of the twentieth century, whose pioneering work left a lasting imprint across neuroanatomy, primatology, and medical education. Born in Hemel Hempstead in 1895, he entered St Thomas' Hospital Medical School at the age of 17 and demonstrated early promise, winning several prestigious awards including the Entrance, William Tite, and Musgrove scholarships (Royal College of Surgeons of England, 2014). His medical training was soon interrupted by the outbreak of the First World War, during which he enrolled as a medical officer in the Royal Army Medical Corps. On the Western Front, Le Gros Clark encountered the grim realities of combat medicine, managing complex traumatic injuries in resource‐limited settings. This early immersion in clinical adversity left a lasting impression, shaping both his pragmatic outlook and his enduring commitment to anatomical precision.

Following the war, he returned to London as an anatomy demonstrator and completed his fellowship examinations, becoming a Fellow of the Royal College of Surgeons (FRCS). His passion for experimental anatomy would flourish in the ensuing years, and in the 1920s, he relocated to Borneo (modern‐day Sarawak, Malaysia) to serve as Principal Medical Officer (Strkalj, 2014). Here, he immersed himself in the study of local primates and undertook pioneering field studies on the spectral tarsier and tree shrew. These early investigations in comparative anatomy contributed not only to the growing field of primate phylogenetics but also provided Le Gros Clark with extensive cross‐disciplinary insight relevant to his clinical practice. His dedication and humility earned him the respect and adoration of the indigenous Sea Dyak people, who famously tattooed him with their tribal insignia as a sign of their appreciation (Strkalj, 2014).

Upon his return to England, Le Gros Clark read anatomy at the University of London and rapidly ascended through several prominent academic posts: first as Professor of Anatomy at St Bartholomew's Hospital (1929–1930); then at St Thomas' Hospital (1930–1934); and finally as Dr. Lee's Professor of Anatomy at the University of Oxford, linked with a fellowship at Hertford College (1934–1962). In 1935, he was elected a Fellow of the Royal Society for his work on primate evolution (Zuckerman, 1973). He also served as Editor of the *Journal of Anatomy* from 1938 to 1945. His work at Oxford signaled a new phase in his career, one focused not only on research and theory, but also on pedagogy and anatomical preservation. Leveraging Oxford's rich skeletal and histological specimens, Le Gros Clark studied primate limb structure and locomotor adaptations in arboreal settings, publishing influential work that shaped mid‐century evolutionary theory (Le Gros Clark, 1934, 1959). Perhaps most famously, he played a central role in debunking the Piltdown hoax in 1953, using careful anatomical analysis to reveal the so‐called Piltdown Man was an artificial chimera of human cranium and orangutan mandible (Straus, 1954). This landmark event underscored his commitment to empirical scrutiny and scientific integrity. His contributions were recognized by election to the American Philosophical Society in 1960 and the award of the Royal Society's Royal Medal in 1961 (Zuckerman, 1973).

During his long and influential tenure at the University of Oxford, Le Gros Clark inherited and expanded a department already steeped in the traditions of neurophysiological inquiry rooted in the legacy of Charles Sherrington yet brought to it a distinctive anatomical sensibility (Molnár & Brown, 2010). Recognizing a rising clinical importance of neurological disease, Le Gros Clark began to focus intensively on the central nervous system (CNS). It was during this period that he curated a remarkable collection of histological and gross neuroanatomical specimens developed for the instruction of Oxford medical students. These preserved slides, now archived in the Department of Physiology, Anatomy & Genetics and available online through the History of Medical Sciences Project website (https://www.medsci.ox.ac.uk/about‐us/history‐of‐medical‐sciences/our‐history/oxford‐medical‐sciences‐through‐the‐centuries/1900s/1900‐1944), constitute a pedagogical archive of unusual depth and diagnostic clarity. His influence is commemorated in the very architecture of the department through the Le Gros Clark building, which housed the Department of Anatomy from 1893 until its closure in 2022.

Le Gros Clark's approach to teaching was as deliberate as his approach to research. In the preface to The Tissues of the Body (1939), the introductory textbook that grew out of his Oxford lectures, he argued that such a work should be not merely informative but also “intellectually stimulating,” and that students should be brought from the earliest stage of their training to vantage points from which the advancing front of anatomical research could be seen (Le Gros Clark, 1939). Anatomy, on this view, was not a settled body of fact to be committed to memory, but an active field of inquiry into which students were to be inducted directly.

The teaching collection examined here should be read in that light. Its composition is weighted not toward normal structure alone, but toward specimens in which a discrete lesion could be mapped onto a specific clinical deficit, and each was accompanied by a descriptive card written in precisely that idiom: an account of what is visible in the section, followed by an account of what the patient would have experienced. Slides were pre‐marked to direct the eye to the relevant field and were arranged in practical classes so that students could move between microscope bays comparing normal and pathological material directly (Box 1). The collection therefore functioned less as an archive than as an instrument of instruction, and its structure encodes an argument about how neuroanatomy should be taught: that anatomical organization is most durably understood through the deficits produced when it fails. That argument has proved unusually persistent, and several of the cases Le Gros Clark selected remain in use in Oxford medical teaching today (Chang & Molnár, 2015).

BOX 1 **Pedagogical use of the Le Gros Clark slide collection in undergraduate medical education.**

Many of the slides in this collection were actively used in undergraduate medical education at the Department of Human Anatomy at the University of Oxford. Practical classes involved setting up half a dozen slides from the Le Gros Clark collection under microscopes in designated bays, accompanied by corresponding descriptive cards. These cards often featured photographs or annotated diagrams to help students correlate microscopic features with functional anatomy. These slides are also scanned and available online: https://learntech.medsci.ox.ac.uk/cslide/items/view/1009. Some slides were pre‐marked with circles or coordinates to guide viewing, allowing students to engage directly with material that illustrated both normal and pathological neuroanatomy. This hands‐on, visual approach reinforced Le Gros Clark's conviction that the teaching of anatomy must be dynamic and conceptually driven. The application of the anatomical knowledge was reinforced by carefully selected clinical cases. The scanned sections of these cases are still used today in neuroanatomy teaching via computer‐assisted teaching material at the University of Oxford.

In this article, we revisit the life and contributions of Le Gros Clark through the lens of this anatomical collection, examining how his integrated approach to structure and function continues to inform the teaching of neuroanatomy. We present previously unpublished archival material that offers new insight into his methodological choices, clinical interests, and academic collaborations. In doing so, we aim to situate his legacy not only within the historical arc of twentieth century anatomy, but also within the ongoing practice of anatomical education.

## 2 METHODS

**FIGURE 1:**
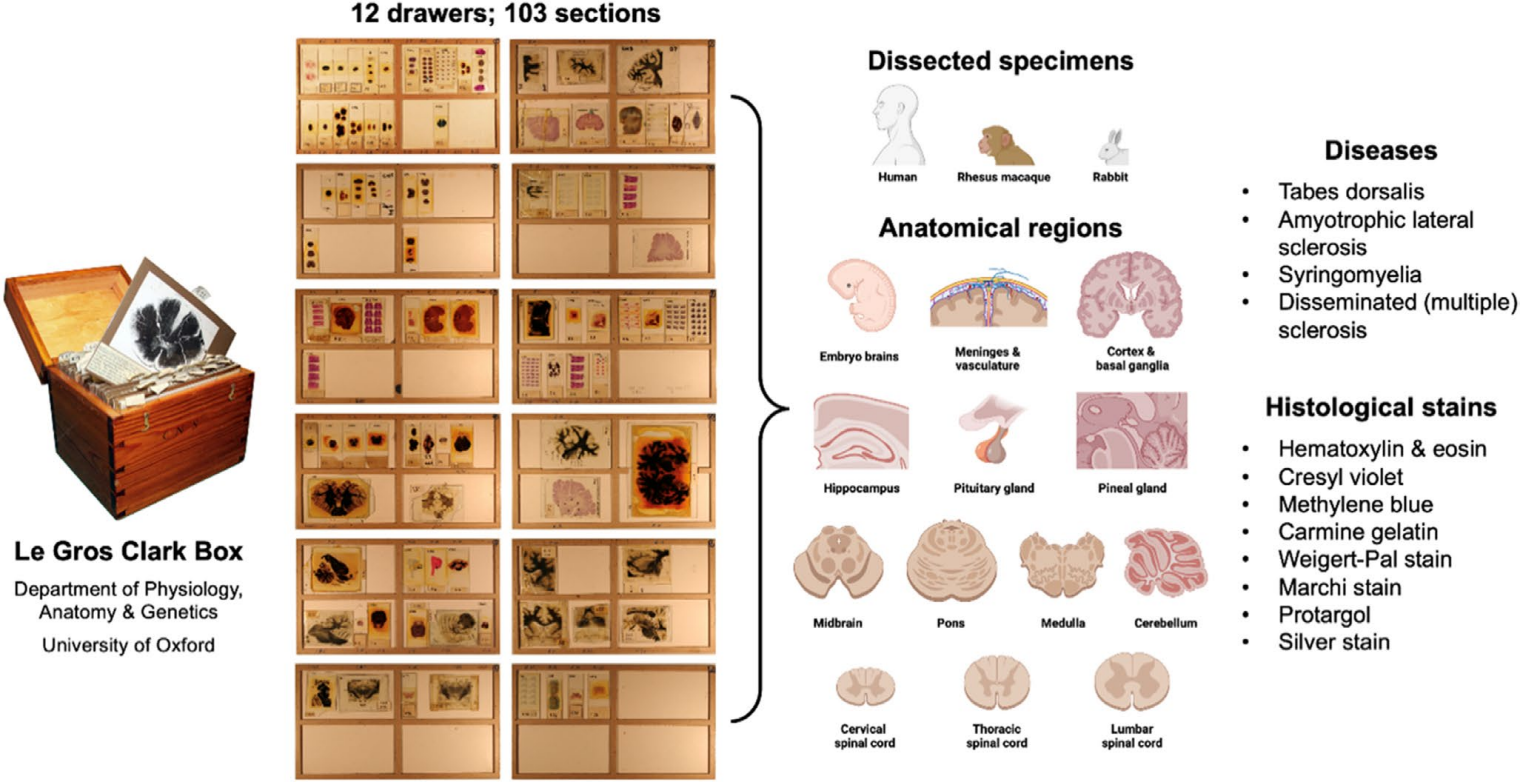
Overview of Le Gros Clark's neuroanatomical teaching collection. Le Gros Clark's teaching collection is archived in a wooden cabinet at the University of Oxford comprising 12 drawers containing a total of 103 histological specimens. The material includes human, rhesus macaque, and rabbit preparations stained using a range of classical histological techniques and encompasses diverse regions of the central nervous system and associated pathological conditions.

The collection of Le Gros Clark's teaching material at the University of Oxford is housed in a large wooden cabinet, with multiple drawers containing meticulously curated anatomical specimens reminiscent of Charles Sherrington's slide box (Molnár & Brown, 2010). For this study, we examined 12 drawers containing a total of 103 specimens (Figure 1), which represent a diverse array of anatomical and pathological preparations used for medical instruction in the mid‐twentieth century. All 103 specimens were examined directly by the authors, together with their accompanying descriptive cards and any annotations present on the slides themselves, and were catalogued by species, anatomical region, staining method, and, where indicated, pathological diagnosis. These specimens were digitized and made available for remote online inspection (https://learntech.medsci.ox.ac.uk/cslide/items/view/1009) via high‐resolution scans (Aperio Digital Pathology Slide Scanner; Leica) of the original histology slides. Further details of how these slides were used in undergraduate medical teaching, including practical class organization and pedagogical annotations, are summarized in Box 1.

Our analysis focused on gross dissections and histological sections derived from human, rhesus macaque, and rabbit material. These included preparations of the embryonic brain, meninges, vasculature, cerebral cortex, basal ganglia, hippocampus, pituitary gland, pineal gland, midbrain, pons, medulla, cerebellum, and spinal cord. Particular attention was paid to diseased specimens with exceptional preservation and histological clarity, including cases of tabes dorsalis, amyotrophic lateral sclerosis, syringomyelia, and disseminated (multiple) sclerosis. No predefined inclusion criteria were applied. Cases were instead selected following review of the collection in its entirety, on the basis of three considerations that emerged from that review: (i) the quality of histological preservation and the clarity with which the relevant pathology could be resolved; (ii) the completeness of the accompanying annotation, such that Le Gros Clark's own clinicopathological reasoning could be recovered alongside the specimen; (iii) the continued anatomical or clinical resonance of the condition for a contemporary readership. The selection was therefore interpretative rather than systematic, and the cases presented here are intended to be illustrative of the collection's pedagogical character rather than statistically representative of its contents. While some of these conditions (such as tabes dorsalis and syringomyelia) are now infrequently encountered in modern clinical practice, others (including multiple sclerosis) remain highly prevalent but rarely available in such well‐preserved histological form. The slides used a range of classic cytoarchitectural and myeloarchitectural staining techniques, including hematoxylin and eosin, cresyl violet, methylene blue, carmine gelatine, Weigert–Pal stain, Marchi stain, protargol, and silver stain (Table 1). Table 1 sets out the principal targets and the resulting appearances of each technique, together with the distinctions between the two Nissl stains and between the two silver methods employed in the collection. Throughout this article, Le Gros Clark's own wording is reproduced verbatim in the figure legends, including his historical terminology, his spinal level notation (e.g., “Th.6” for the sixth thoracic segment), and period spellings such as “demyelinization.” Where these differ from current usage, the modern equivalent is given at first occurrence in the main text. We use dorsal and ventral in our descriptions and retain anterior and posterior where they appear in his annotations—in the human spinal cord these pairs are equivalent.

**TABLE 1:** Histological staining techniques and their anatomical targets in the Le Gros Clark teaching collection.

Stain	Target	Appearance
Hematoxylin and eosin	Hematoxylin: basophilic components (DNA/RNA) Eosin: eosinophilic components (cytoplasm, collagen, extracellular protein)	Hematoxylin: blue to purple Eosin: pink to red, with erythrocytes bright red
Cresyl violet^a^	Nissl substance (rough endoplasmic reticulum/ribosomes, particularly in the RNA‐rich cytoplasm of neurons), neuronal and glial nuclei	Violet to deep purple against a pale, largely unstained neuropil
Methylene blue^a^	Basophilic structures (nuclei, Nissl substance). Historically also used to supravitally demonstrate peripheral nerve fibers and endings	Blue, with less differentiation between Nissl substance and background than Cresyl violet
Carmine gelatine	Glycogen, mucins, cell nuclei	Bright crimson red
Protargol (silver proteinate)^b^	Neuronal perikarya and processes (axons, dendrites, neurofibrils). Impregnates uniformly across the neuronal population	Dark brown to black neuronal elements against a golden‐brown background
Silver impregnation^b^	Neurofibrils and axons, argyrophilic reticular fibers, basement membranes	Black against a yellow to pale brown background
Weigert–Pal^c^	Myelin sheaths of axons. Demyelinated regions remain unstained	Dark blue‐black myelin against pale, unstained demyelinated tissue
Marchi^c^	Degenerating/denatured myelin sheaths. Normal myelin remains unstained	Black droplets and granules of degenerate myelin against an unstained background

*Note*: This table summarizes the classical histological staining methods employed across the Le Gros Clark neuroanatomical teaching collection, together with their principal cellular and tissue targets. The stains reflect mid‐twentieth century pedagogical practice and were selected to emphasize cytoarchitectural, myeloarchitectural, and fiber‐tract organization within both normal and pathological specimens of the central nervous system. Collectively, these techniques underpin the clinicopathological interpretations illustrated in Figures 2, 3, 4, 5, 6 and exemplify the methodological foundations of neuroanatomical teaching during Le Gros Clark's tenure at the University of Oxford.

^a^ Cresyl violet and methylene blue are both basic aniline dyes that bind nucleic acids, and their targets therefore overlap substantially. They differ chiefly in selectivity and application: Cresyl violet is the standard Nissl technique and yields crisp, well‐differentiated staining of Nissl substance suited to cytoarchitectural analysis, whereas methylene blue stains the same basophilic structures with less differentiation and was historically also employed as a supravital stain for peripheral nerve fibers.

^b^ Protargol and the general silver impregnation methods likewise share a common chemistry but differ in selectivity: protargol employs a preformed silver–protein complex that impregnates neurofibrils reproducibly and uniformly throughout the neuronal population, whereas conventional silver methods deposit silver from free silver nitrate and are correspondingly more variable, staining either a sparse and randomly distributed subset of neurons in their entirety or, in reticulin methods, argyrophilic fibers and basement membranes rather than neurons.

^c^ Weigert–Pal and Marchi are complementary in logic: the former stains intact myelin, so that pathology appears as an absence of staining, while the latter stains only degenerate myelin, so that pathology appears as a positive signal.

In addition to the slide collection, we examined a series of previously unpublished letters and archival materials associated with Le Gros Clark. This correspondence provides valuable insight into the historical context, intellectual influences, and curatorial decisions underlying the collection's assembly. These materials were read as contextual source documents rather than analyzed within a formal historiographical framework. They are used here to situate the collection within Le Gros Clark's wider academic activity and to corroborate details of its assembly and use, and we make no claim of systematic archival analysis.

### 2.1 Tracing the sclerotic plaque: A mid‐century perspective on disseminated (multiple) sclerosis

Disseminated (or multiple) sclerosis is a chronic inflammatory disease of the CNS, whose original description (“*la sclérose en plaques disseminées*”) is classically attributed to Charcot (Zalc, 2018). Early translations of this phrase include “disseminated sclerosis” and “multiple sclerosis” when referring to the disease, with the former term being preferred in the United Kingdom until the 1950s, after which “multiple sclerosis” became standard nomenclature (Talley, 2004). Le Gros Clark's annotations date before this transition and use “disseminated sclerosis” throughout—we follow his usage when discussing his material and modern usage when discussing the disease as currently understood.

Owing to the pioneering work of Rindfleisch, Dawson, and others, the pathology of disseminated sclerosis was rather well‐characterized and largely consolidated by the 1930s (Lassmann, 1999), around the time Le Gros Clark began teaching at Oxford. Seminal pathologic studies had established the “sclerotic plaque,” a circumscribed patch of demyelination with relative axonal preservation and a reactive glial scar, as the pathological hallmark of the disease. Importantly, these plaques were repeatedly linked to the microvasculature, with a small vein often noted at the lesion epicenter—an insight that anticipated the modern paradigm of perivenular demyelination. Dawson's meticulous histological monograph (Dawson, 1916) subsequently cemented this topology and the inflammatory nature of lesions, describing the now‐classic picture of perivenular cuffs and extension along venous territories, as well as periventricular plaque concentration due to involvement of the subependymal veins.

By 1930, Brain's influential review (Brain, 1930) explicitly framed plaques as evolving structures: first as an “early patch” characterized by vasodilation, prominent inflammatory infiltration and tissue injury with concurrent demyelination (now termed an “active lesion”), progressing to a “late patch” where inflammation subsides and the plaque becomes dominated by gliosis and vascular changes (now termed an “inactive lesion”). Although these accounts focused heavily on white matter changes, plaques were also noted to extend into the cortical and deep gray matter, but the significance of this observation was not fully appreciated until later (Calabrese et al., 2015). Contemporary work has since implicated gray matter pathology as a key determinant of clinical disability and disease severity (Geurts et al., 2012).

**FIGURE 2:**
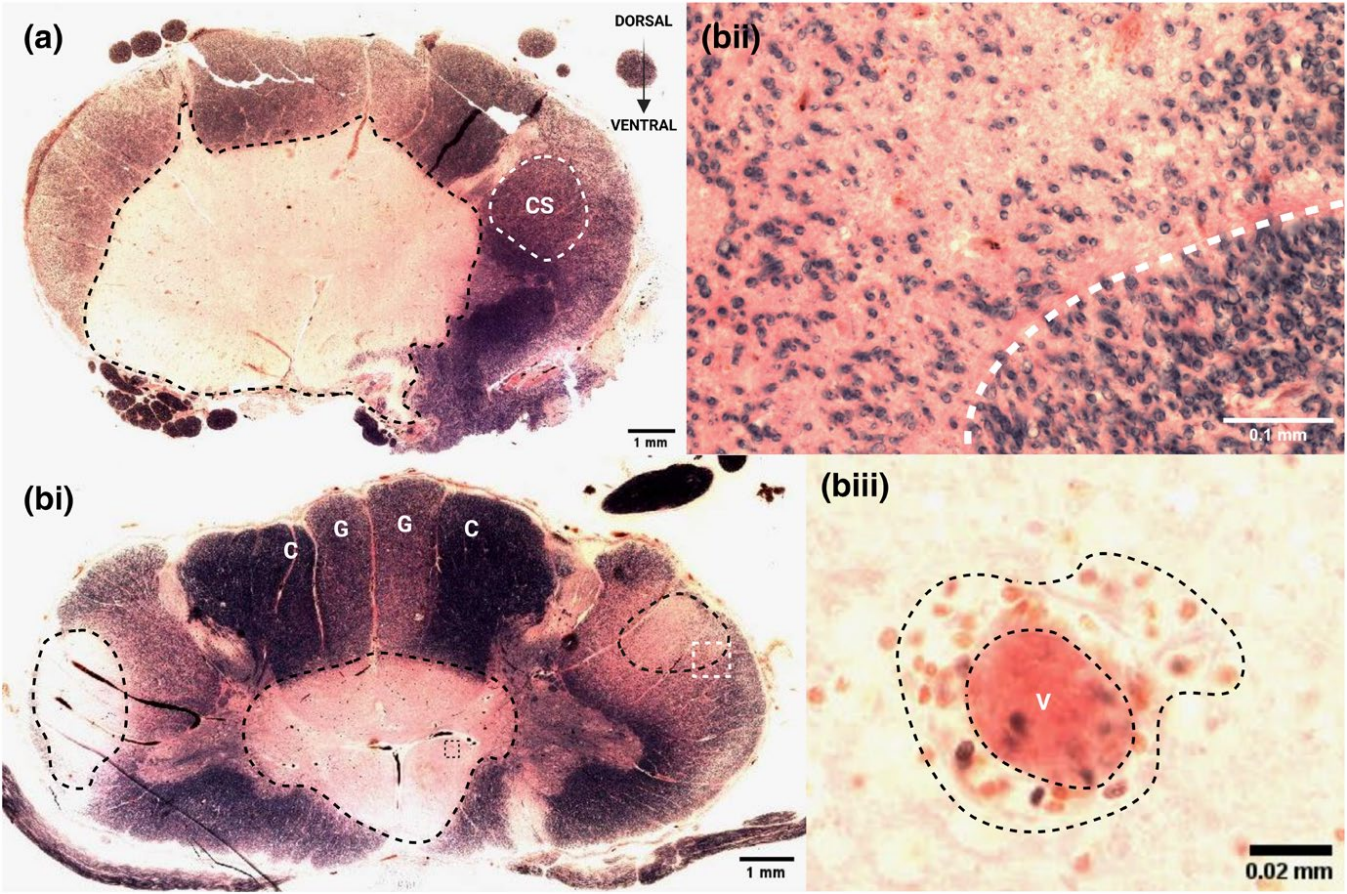
Disseminated (multiple) sclerosis in the human brainstem and spinal cord (Weigert–Pal stain with red counterstain). (a) Transverse section of the medulla showing a large demyelinating plaque (outlined) that occupies much of the section and encroaches on the corticospinal tract (CS), indicated on one side where its boundary could be identified with confidence. (bi) Transverse section of the cervical spinal cord section demonstrating multiple discrete plaques (outlined) involving the lateral columns bilaterally and extending into the central gray matter, consistent with Le Gros Clark's observation that the lesions were not confined to any definite tract. The posterior columns are comparatively preserved and are divided into the *fasciculus gracilis* (G) medially and the *fasciculus cuneatus* (C) laterally. The boxed regions in B are shown in the panels to the right. (cii) The plaque border (dashed line) showing an abrupt reduction in myelinated axon density across the lesion boundary. (biii) A blood vessel (BV) within the plaque, surrounded by a cuff of mononuclear inflammatory cells (outline), demonstrating the perivascular distribution of the infiltrate. Dorsal is uppermost in all panels, as indicated in panel a. G, *fasciculus gracilis*; C, *fasciculus cuneatus*; CS, lateral corticospinal tract; V, blood vessel. Scale bars: 1 mm (a, b), 0.1 mm (bi), 0.02 mm (bii). Notes from Le Gros Clark: “Sections of the medulla (Relating to Figure a) and of the cervical region of the spinal cord (Relating to Figure bi) from a case of disseminated sclerosis. They show plaques of degeneration (or patches of demyelinization) involving different parts and not confined to any definite tract and also affecting the gray matter. All the long tracts of the cord become damaged to varying degrees, the motor systems being most affected. Thus, the patient shows the effects of pyramidal tract interruption (spastic paralysis) as well as cerebellar ataxia. The combination of these two commonly leads to the development of an intention tremor (a coarse trembling of muscles which are taking part in a voluntary movement). Disseminated sclerosis is characterized by attacks of severe paralysis affecting one or more limbs, followed by partial recovery. The attacks recur, and each time leave the patient in a worse condition. The progress of the disease is relatively slow, and even after 10–15 years, the disability may still not be very severe.”

Here, we present medullary and cervical spinal cord sections from a patient with disseminated sclerosis (Figure 2a,b), stained using the Weigert–Pal method (Bolon et al., 2025) to highlight myelinated fibers. In the medulla, a single large plaque is present anteromedially, involving the pyramids bilaterally. Similarly, in the cervical cord, there is a large plaque surrounding the central canal and gray matter, in addition to significant demyelination of the lateral columns. At the plaque boundary, there is a marked reduction in the density of myelinated axons (Figure 2bi), and within plaques, the inflammatory infiltrate is seen to concentrate around the microvasculature (Figure 2bii).

Disseminated sclerosis can manifest with both sensory and motor deficits, depending on the localization of plaques. Le Gros Clark reports that this individual suffered spastic paralysis owing to disruption of the pyramidal tract and intention tremor, possibly reflecting involvement of the spinocerebellar tracts or another cerebellar pathway not illustrated here.

Collectively, these early foundations remain highly relevant in our current understanding of the pathology of disseminated sclerosis. The pathology and symptomatology are highly consistent with this diagnosis and in many ways represent a “typical” case.

### 2.2 Degeneration of the motor system: Amyotrophic lateral sclerosis and progressive muscular atrophy

Amyotrophic lateral sclerosis (ALS) is a progressive, fatal disease defined clinically by mixed upper motor neuron (UMN) and lower motor neuron (LMN) dysfunction, and anatomically by degeneration of the corticospinal tracts, spinal, and bulbar motor neurons (Feldman et al. 2022). Although its first description has been credited to Charles Bell, Charcot's lasting contribution (Rowland, 2001) was to formalize ALS as a distinct clinicopathological entity via rigorous mapping of neurological signs onto discrete lesions, exemplifying “*la méthode anatomo‐clinique*” (‘anatomo‐clinical method’) (Goetz, 2010).

Charcot's synthesis emerged from a series of studies in the nineteenth century through which he and colleagues separated a mixed motor syndrome from other progressive muscular atrophies. In 1865, he famously presented a case report at the Société Médicale des Hôpitaux de Paris, describing a young woman with progressive but profound weakness and increased muscle tone—at autopsy, pathological evaluation revealed degeneration confined to the lateral columns. A second key advance came in 1869 from analyses of pediatric cases of infantile paralysis, in which lesions were found to be restricted to the anterior gray horns, and patients exhibited muscle atrophy. Combining these observations, Charcot deduced that the motor system of the spinal cord was organized in two parts, with each division possessing a distinct anatomical lesion (Goetz, 2000), a framework that remains embedded in modern diagnostic reasoning.

In subsequent lectures published in 1874, Charcot consolidated these concepts under the term “*sclérose latérale amyotrophique*,” explicitly dividing the muscular atrophies into the “true spinal” muscular atrophies (“*atrophie musculaire progressive*”) caused by loss of anterior horn cells, and the “false spinal” muscular atrophies resulting from secondary loss of anterior horn cells and associated with degeneration of the lateral funiculi (“*la sclérose latérale amyotrophique*”) (Corcia & Meininger, 2019). Much debate ensued over the following decades as to whether progressive muscular atrophy (PMA) truly represented a distinct entity or was simply a variant of ALS, with many neurologists accepting the latter, integrated view (Visser et al., 2008).

Reflecting the fact that mixed UMN/LMN presentations dominate clinical practice, the terms “ALS” and “motor neuron disease” (MND) were often applied interchangeably (Bäumer et al., 2014). Contemporary nosology instead recognizes ALS as the most common phenotype within a broader MND spectrum, which includes UMN‐predominant primary lateral sclerosis (PLS) and LMN‐predominant PMA, as well as bulbar‐onset variants historically framed as progressive bulbar palsy (PBP). This phenotypic framing is underpinned by the observed neuropathological overlap between these disorders, with frequent evidence of “hidden” UMN involvement in ostensibly LMN‐restricted disease (Ince et al., 2003).

**FIGURE 3:**
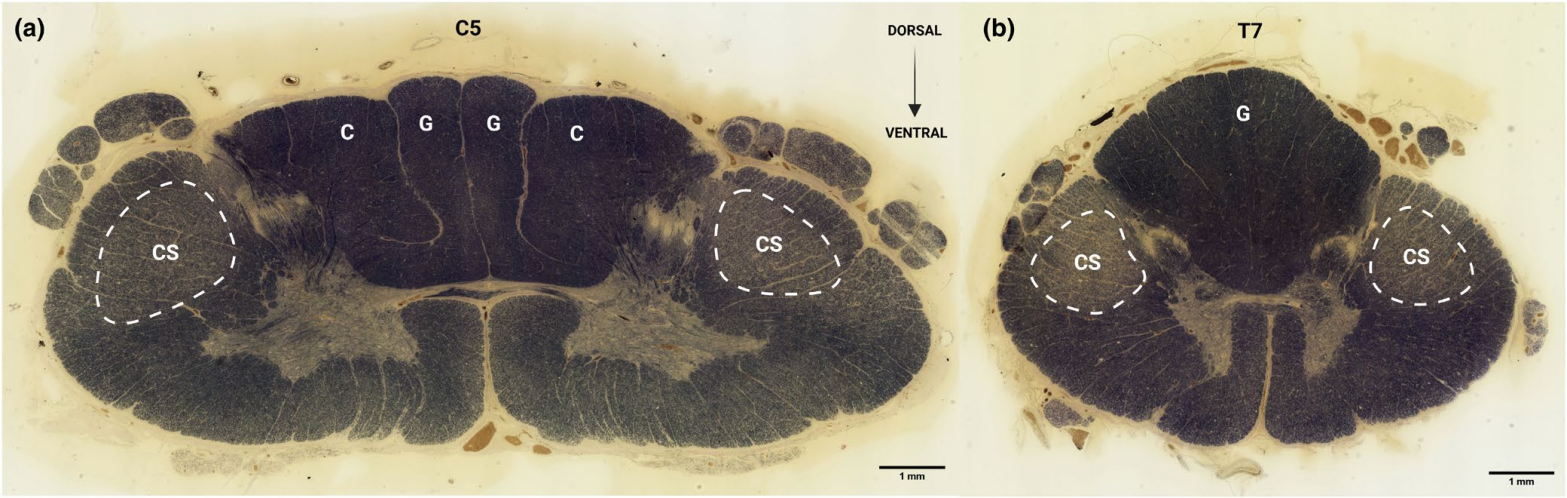
Progressive muscular atrophy (amyotrophic lateral sclerosis) in the human spinal cord (Weigert–Pal stain). (a) Transverse section of the spinal cord at C5 level demonstrating bilateral degeneration of the lateral corticospinal tracts (outlined). The posterior columns are divided into the *fasciculus gracilis* (G) medially and the *fasciculus cuneatus* (C) laterally. (b) Transverse section at T7 level showing continued corticospinal tract degeneration—below T6 the posterior funiculus comprises the *fasciculus gracilis* (G) alone. In both sections, the posterior columns demonstrate relative preservation with increased staining intensity, an appearance Le Gros Clark noted as characteristic of the condition for which he could offer no explanation. Dorsal is uppermost in both panels, as indicated in panel a. G, *fasciculus gracilis*; C, *fasciculus cuneatus*; CS, lateral corticospinal tract. Scale bars: 1 mm. Notes from Le Gros Clark: “Sections of the spinal cord at levels C.5 (Relating to Figure a) and Th. 7 (Relating to Figure b) from a case of progressive muscular atrophy (amyotrophic lateral sclerosis) showing degeneration of the pyramidal tracts. This disease also affects the motor cells in the anterior horns—many of which degenerate and disappear. In other words, there is a combined degeneration of upper and lower motor neurons. The patient at first shows a progressive spastic weakness of the lower limbs, associated with a gross wasting of the muscles (due to the lower motor neuron lesion). Later on, this wasting is often best seen in the upper limbs. The wasted muscles show peculiar irregular twitches (fasciculation). There is no sensory loss in this condition. Note the dark staining of the posterior columns of the spinal cord; this is a characteristic appearance in cases of progressive muscular atrophy, and the reason for it is not clear.”

Here, we present two spinal cord sections stained using the Weigert–Pal method (Figure 3). Although the case is labelled “progressive muscular atrophy,” the marked degeneration of the pyramidal tracts indicates substantial UMN involvement, which instead points toward a mixed disorder (ALS). However, in light of the historical context, Le Gros Clark's labelling reflects the nosological usage of the period, in which the terms PMA and ALS were often applied interchangeably, as reflected by the fact that both terms appear in his original case description (Figure 3).

### 2.3 Tabes dorsalis: The disappearing disease preserved in the dorsal columns

Tabes dorsalis is a late manifestation of tertiary syphilis, marked by selective demyelination of the dorsal columns and posterior nerve roots of the spinal cord. Its clinical and pathological features were first described in the nineteenth century, with key contributions by Duchenne, Romberg, and ultimately Charcot, who provided a comprehensive account of “*ataxie locomotrice progressive*” (progressive locomotor ataxia) and linked it to spinal cord pathology (Tatu & Bogousslavsky, 2021). Around this time, the pathognomonic sign of neurosyphilis, the Argyll Robertson pupil, was also described (Robertson, 1869a, 1869b).

For a long time, the term “*tabes*” (from Latin: “consumption,” “wasting away”) was used in medicine to denote consumption of various origins, such as the lungs (*tabes pulmonalis*) and nervous system (*tabes nervosa*) (Tatu & Bogousslavsky, 2021). It is unclear when the specific term “*tabes dorsalis*” emerged, but it was probably in the seventeenth century (Olry & Haines, 2018). Much confusion surrounded the nineteenth century, since terms such as “*tabes dorsalis*,” “progressive locomotor ataxia,” and “*tabes dorsal ataxique*” (ataxic dorsal tabes) were all being used inconsistently to describe the same disease.

**FIGURE 4:**
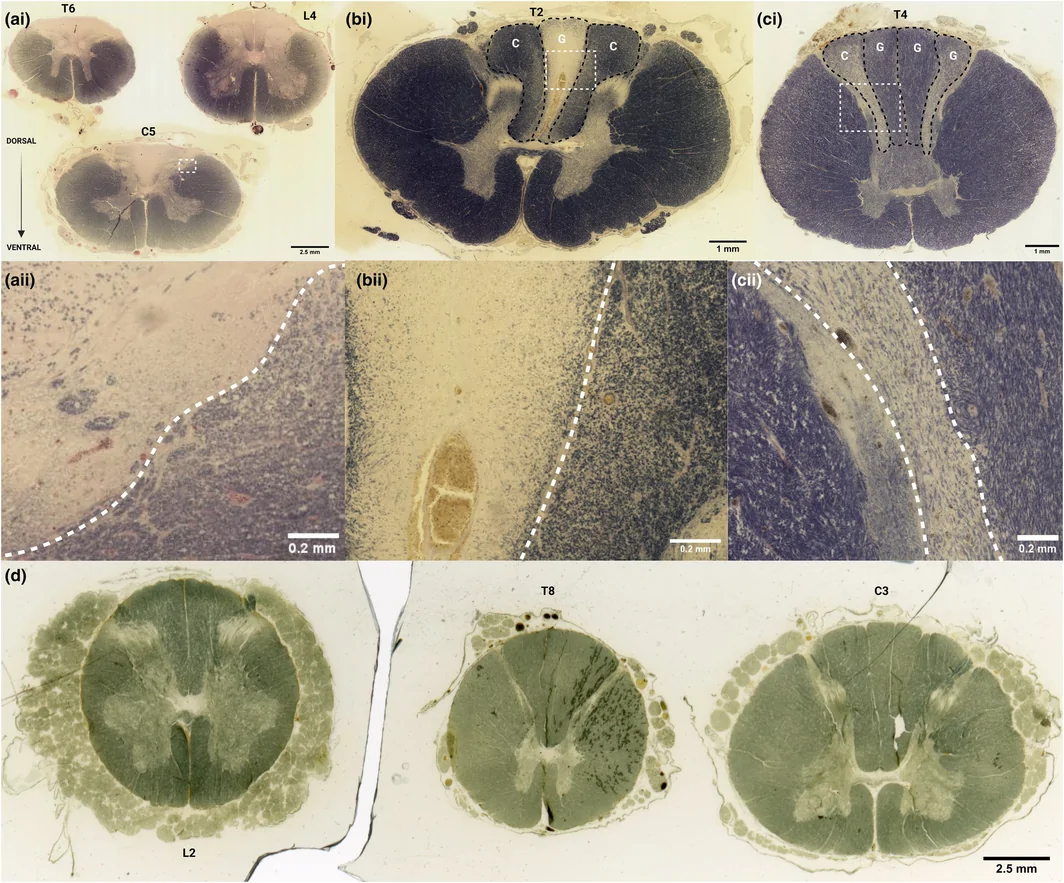
Tabes dorsalis in the human spinal cord (Weigert–Pal stain). (ai) Transverse sections of the spinal cord at C5, T6, and L4 levels from a patient with tabes dorsalis showing bilateral demyelination of the dorsal columns involving both the *fasciculus gracilis* and *fasciculus cuneatus*. The boxed region in the C5 section is shown in the panel below. (aii) The lesion border (dashed line) marking the transition between demyelinated dorsal column tissue (pale) and preserved white matter (dark). (bi) Transverse section at T2 level from a second patient demonstrating selective demyelination of the *fasciculus gracilis* (G) with relative sparing of the *fasciculus cuneatus* (C). The boxed region is shown in the panel below. (bii) The border between the demyelinated gracile territory and the preserved cuneate territory (dashed line). (ci) Transverse section at T4 from a case of ‘cervical tabes’ showing isolated bilateral demyelination of the *fasciculus cuneatus* (C) with sparing of the *fasciculus gracilis* (G). The boxed region is shown in the panel below. (cii) The cuneate‐gracile border (dashed lines) at higher resolution, with the demyelinated cuneate territory between them. (d) Transverse sections from a third patient at L2, T8, and C3 (left to right, as annotated on the slide), demonstrating a more subtle pattern of posterior column degeneration. Dorsal is uppermost in all panels, as indicated in panel ai. G, *fasciculus gracilis*; C, *fasciculus cuneatus*. Scale bars: 2.5 mm (ai, d), 1 mm (bi, ci), 0.2 mm (aii, bii, cii). Notes from Le Gros Clark: (Relating to Figure ai, aii “Section through the spinal cord (from levels C.5, Th.6, and L.4) from a patient suffering from tabes dorsalis. They show almost complete degeneration of both posterior column tracts on either side (tracts of Goll and Burdach). The usual signs and symptoms of this disease include loss of sense of position and passive movement, of tactile discrimination and localization, and of vibration sense. The motor tracts are unaffected, and thus there would be no actual paralysis of the voluntary musculature.” (Relating to Figure bi, bii) “Section of the spinal cord (at the level of Th.2) from a patient suffering from tabes dorsalis and showing degeneration of the tract of Goll (*fasciculus gracilis*). This tract conveys proprioceptive impulses from the lower extremity. The patient would have shown a typical ataxic gait, with loss of postural sense and tactile discrimination in the lower limbs. There would also be loss of vibration sense and ability to localize tactile stimuli. On the other hand, there would be no weakness or paralysis of the muscles of the lower limb.” (Relating to Figure ci, cii) “Section of the spinal cord (at the level of Th.4) showing degeneration of the tract of Burdach (*fasciculus cuneatus*), a degeneration which extends into the cervical region of the cord. This rare condition is called “cervical tabes” and, since the tract of Burdach conveys proprioceptive impulses from the upper limb, it leads to a loss of postural sense, tactile discrimination and localization, and vibration sense in the upper limbs. The patient would have difficulty in carrying out fine manipulations accurately. He would also lose the discriminative sensibility of his fingers and if, for example, he placed his hand in his pocket, he would not be able to pick out one coin from another just by the sense of touch.” (Relating to Figure d) “These are also sections of the spinal cord from a tabetic patient, at levels C.3, Th.8, and L.2. The degeneration of the posterior column tracts is not so marked as in the previous (Relating to Figure ai) specimen.”

By the mid‐twentieth century, however, tabes dorsalis was widely recognised as a distinct spinal cord syndrome caused by the spirochete *Treponema pallidum* and characterized by distinct anatomical features, notably degeneration of the fasciculi *gracilis* and *cuneatus* with relative sparing of motor tracts. These tracts were known throughout the nineteenth and early twentieth centuries as the tracts of Goll and Burdach, respectively, and it is by these names, Le Gros Clark refers to them in his annotations. Classic neuropathological studies emphasized a lumbosacral predominance, with several documented cases of selective *fasciculus gracilis* involvement (Davison & Kelman, 1937) (Figure 4b). However, Ferrier noted that in certain cases, pathology may be restricted to the *fasciculus cuneatus* in the cervical region (so‐called “cervical tabes”) (Ferrier, 1906) (Figure 4c).

A long‐standing neuropathological model was proposed by Obersteiner and Redlich, who described the normal structure of the dorsal roots and dorsal columns, and postulated that tabetic degeneration begins at a “*locus minoris residentiae*” where the root acquires meningeal coverings (the so‐called Obersteiner–Redlich zone) and might be most vulnerable to inflammatory or toxic influences (Savvaidou & Triarhou, 2016). Early mechanistic syntheses (including those of Schaller and Adie) framed tabes dorsalis as an active syphilitic leptomeningitis or meningoradiculitis, in which chronic meningeal inflammation preferentially injures the posterior roots at their entry zones, with dorsal column degeneration arising secondarily via the central processes of dorsal root ganglion neurons (Adie, 1921; Schaller, 1919). This model naturally suggests an ascending progression: lumbosacral afferents encompass the longest ascending fibers and may be most vulnerable to an initial segmental lesion, possibly accounting for the apparent lumbosacral predilection of tabes dorsalis.

Nevertheless, the pathogenesis remains poorly understood: contemporary reviews describe tabes as a slowly evolving process in which direct spirochete invasion and/or dysregulated host immune responses are both biologically plausible contributors to neuronal injury (Carlson et al., 2011). These changes are closely associated with wide‐based ataxic gait, lancinating pains, loss of vibration sense, and diminished deep tendon reflexes, all secondary to disruption of dorsal column‐mediated proprioception (Schiller, 1995).

Although syphilis rates plummeted after the advent of penicillin, and decreased further in the 1990s due to more effective treatment paradigms and societal change (Kojima & Klausner, 2018), its incidence has paradoxically risen in recent years (Fountain et al., 2024). Syphilis is notably regarded as a “great mimicker” owing to its widely variable presentation, particularly in the secondary stage (Fenner et al., 2025), and cases of tabes dorsalis continue to be reported (Bloch et al., 2025). Historical cases (such as those in the Le Gros Clark collection) thus remain critically relevant for the modern medical student.

Here, we present transverse spinal cord sections from four patients with tabes dorsalis, stained using a myelin‐specific technique to visualize dorsal column degeneration. In the first case (Figure 4ai), there is severe, bilateral demyelination of both dorsal column tracts at cervical, thoracic, and lumbar levels. Within the boxed region (Figure 4aii), a distinct boundary between preserved and demyelinated tissue is visible, with sparse surviving myelinated axons amidst widespread demyelination.

The second case (Figure 4bi) shows selective demyelination of the *fasciculus gracilis* with relative preservation of the *fasciculus cuneatus*, consistent with impaired lower limb proprioception but intact upper limb proprioception. At the gracile‐cuneate border (Figure 4bii), the degenerate gracile territory is markedly pale against the adjacent preserved cuneate fibers.

The third case (Figure 4ci) illustrates the converse pattern, with selective demyelination of the *fasciculus cuneatus* bilaterally and sparing of the gracile fibers—the rare presentation Le Gros Clark termed “cervical tabes”—suggesting proprioceptive disturbance confined to the upper limbs; the cuneate‐gracile border is shown in Figure 4cii. A fourth case (Figure 4d) demonstrates a more subtle pattern of degeneration than the preceding examples, with only partial pallor of the dorsal columns.

These preserved slides not only illustrate the classical neuroanatomy of a once feared (and now rare) condition, but also serve as enduring artefacts of a bygone clinical era, reminding us that even a disappearing disease can leave lasting traces in both the spinal cord and the annals of medical education.

### 2.4 The pipe within: Syringomyelia through time and tissue

Syringomyelia, derived from the Greek *syrinx* meaning “pipe” or “tube,” refers to the formation and progressive expansion of an intramedullary cystic cavity most often in the cervical spine. Although the term “syringomyelia” was coined in the early 1800s by the French pathologist Charles‐Prosper Ollivier d'Angers and marked a pivotal moment in the nosology of spinal disorders, the anatomical basis of syringomyelia was noted centuries earlier during the Renaissance era (Mortazavi et al., 2011). In *La dissection des parties du corps humain* (1543), Charles Estienne provided the first anatomical illustrations of longitudinal spinal cord cavities distinct from the central canal, though these early observations lacked clinical correlates. Later, Johann Conrad Brunner (1688) and Giovanni Morgagni (1761) documented similar observations (Demetriades, 2012). By the late 1800s, Schüppel articulated a key distinction between hydromyelia (ependymal‐lined dilatation of the central canal) and true syringomyelia (glial‐lined cavity separate from the canal) (Schüppel, 1865). In 1882, von Kahler and Schultze linked the disease to muscular atrophy and the characteristic dissociation of pain and temperature loss. Building on these foundations, Jackson & Lockhart Clarke's landmark 1867 paper correlated pathological cavitation with progressive upper limb wasting and segmental neurological deficits, and would have been well known to anatomists of Le Gros Clark's generation (Ramachandran & Aronson, 2012).

**FIGURE 5:**
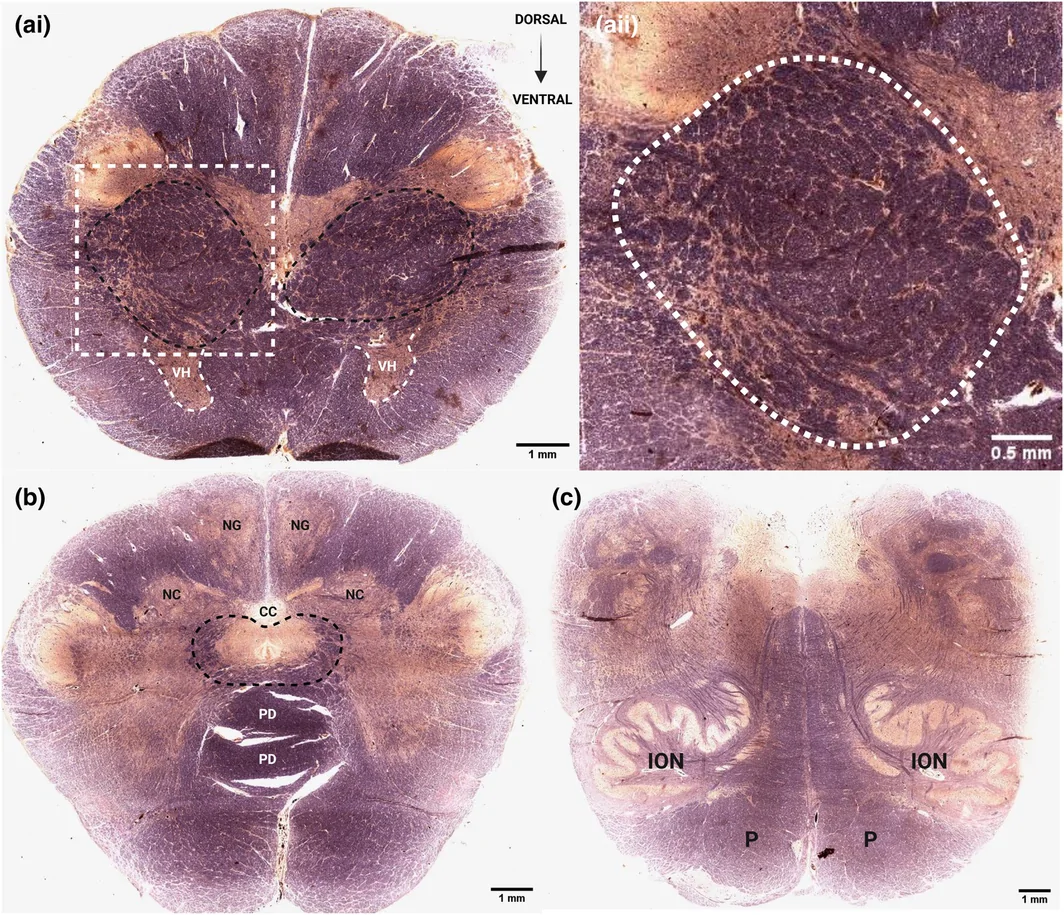
Presumed syringomyelia in the human cervical spinal cord and brainstem (Weigert–Pal stain). (ai) Transverse section of the cervical spinal cord section demonstrating extensive cystic degeneration of the central region (outlined). The destruction encompasses the territory of the central canal and anterior white commissure, neither of which remains identifiable as a discrete structure, and extends laterally into both ventral horns (VH). The boxed region is shown in the panel to the right. (aii) The cavitated central zone (outlined), showing loss of normal gray and white matter architecture. (b) Section through the spinomedullary junction illustrating rostral extension of the degenerative changes (outlined) surrounding the central canal (CC), with the *nucleus gracilis* (NG) and *nucleus cuneatus* (NC) preserved dorsally, and the pyramidal decussation (PD) visible ventrally. (c) Section through the medulla at the level of the inferior olivary nuclei (ION), representing the rostral extent of the preserved specimen series. No discrete cavitation is present at this level. Dorsal is uppermost in all panels. VH, ventral horn; CC, central canal; NG, *nucleus gracilis*; NC, *nucleus cuneatus*; ION, inferior olivary nucleus; P, pyramid; PD, pyramidal decussation. Scale bars: 1 mm (ai, b, c), 0.5 mm (aii). Notes from Le Gros Clark: “Section of the cervical region of the spinal cord from a case of syringomyelia. In this condition, a cystic degeneration begins in the region of the central canal and extends upward and downward in cylindrical form along a length of the cord, involving a number of segments. As it spreads out peripherally from the central canal, it involves quite early the anterior commissure of the cord, interrupting the spinothalamic tracts which are decussating here. Consequently, it leads to bilateral loss of pain and temperature sensibility over a corresponding number of segments in the body. In later stages, the degenerative process extends out to the anterior horns, destroying the anterior horn cells (and thus leading to a wasting of the limbs). Still later, it may involve the pyramidal tracts, and posterior column tracts, with a corresponding extension of motor and sensory disturbances. In this case, the condition affected the cervical region of the cord, and in its early stages led to a loss of pain and temperature sensation in both upper limbs. As the section shows, the disease became very extensive before the patient died, destroying the whole of the central region of the cord.”

Le Gros Clark's preserved cervical spinal cord specimen (Figure 5) aligns closely with this evolving narrative. While a discrete syrinx is not discernible in the available histological sections, the observed pattern of degeneration (central gray matter atrophy particularly affecting the anterior horns and anterior white commissure) strongly supports a presumptive diagnosis of syringomyelia (Figure 5a). The mild longitudinal expansion (Figure 5b,c) raises the possibility of a collapsed or resolved syrinx, or gliotic degeneration following prior cavitation. Le Gros Clark's annotation makes no reference to antecedent trauma, and describes the lesion in the classical terms of central cavitation spreading outward from the region of the central canal. We note, however, that his wartime experience of spinal injury would have made him familiar with trauma‐related myelopathy, and the appearances in this specimen are of interest in light of what is now recognized as post‐traumatic syringomyelia—a connection we draw retrospectively rather than one he proposed.

Though formally characterized in the 1960s, post‐traumatic syringomyelia has been intermittently described in the literature. Greitz (Greitz, 2006) later refined its pathophysiological framework, proposing that spinal trauma results in segmental venous hypertension and cerebrospinal fluid flow impedance, initiating syrinx formation through pressure differentials following initial traumatic insults. Other proposed mechanisms include arachnoid scarring, altered cerebrospinal fluid pulse transmission, and microvascular ischemia, each contributing to impaired perivascular fluid clearance and pressure dissociation between subarachnoid and intramedullary compartments. Post‐traumatic syringomyelia is estimated to occur in approximately 3%–8% of patients with spinal cord injuries, though it may present years or even decades after the initial event, often with insidious symptom progression (Wu et al., 2023). The cervical localization of degeneration in Le Gros Clark's specimen is consistent with regions vulnerable to flexion–extension trauma and corresponds clinically with areas associated with the highest incidence of symptom onset, particularly with segmental arm weakness, cape‐like pain and temperature loss, and hand muscle wasting, all of which were noted in Le Gros Clark's specimen annotations. Notably, later MRI studies have shown that up to 30% of post‐traumatic syringomyelia patients may exhibit irregular, discontinuous, or non‐cystic cavitation, and histopathological correlates often reveal segmental cystic degeneration even in the absence of a discrete syrinx (Biyani & Masry, 1994). In this context, Le Gros Clark's presumptive diagnosis is therefore well supported by the appearances, and the absence of a discrete syrinx, which might otherwise be taken to argue against it, is consistent with what is now known of segmental cystic degeneration in chronic disease.

Le Gros Clark's specimens thus stand as an early example of clinicopathological deduction in the absence of neuroimaging. Today, classification systems based on MRI morphology help distinguish communicating and noncommunicating syringomyelia, though this insight from neuroimaging was nonexistent during Le Gros Clark's era, highlighting his reliance on purely anatomical deduction. His corresponding annotations, which detail dissociated sensory loss in the upper limbs followed by motor decline, map precisely onto the affected anatomical regions in the spinal cord. While modern classification now distinguishes between congenital, neoplastic, idiopathic, and post‐traumatic forms of syringomyelia, the core anatomical mechanism of central cord destruction with CSF‐mediated pathophysiology remains the same (Wisoff, 1988). Le Gros Clark's archival material therefore continues to offer valuable insight into the anatomical and historical trajectory of another complex neurological disease process, with continued relevance in the modern era.

### 2.5 Unravelling the *nervus conarii*: From anatomical curiosity to circadian pathway

In the 1930s and 1940s, Le Gros Clark devoted a considerable amount of anatomical and histological effort to the pineal gland and its surrounding structures, motivated by an enduring curiosity about a fiber bundle previously described as the *nervus conarii*, and which he refers to in his own teaching annotations, and in publications, simply as the “pineal nerve” (Le Gros Clark, 1940a). First identified in mammals by Kolmer and Löwy (1922), the *nervus conarii* was thought to emerge from the pineal gland and travel along the great cerebral vein of Galen. This pathway intrigued Le Gros Clark, who meticulously traced the fiber bundle in both human and rhesus macaque and published his findings in a series of anatomical notes (Le Gros Clark, 1940a, 1940b).

**FIGURE 6:**
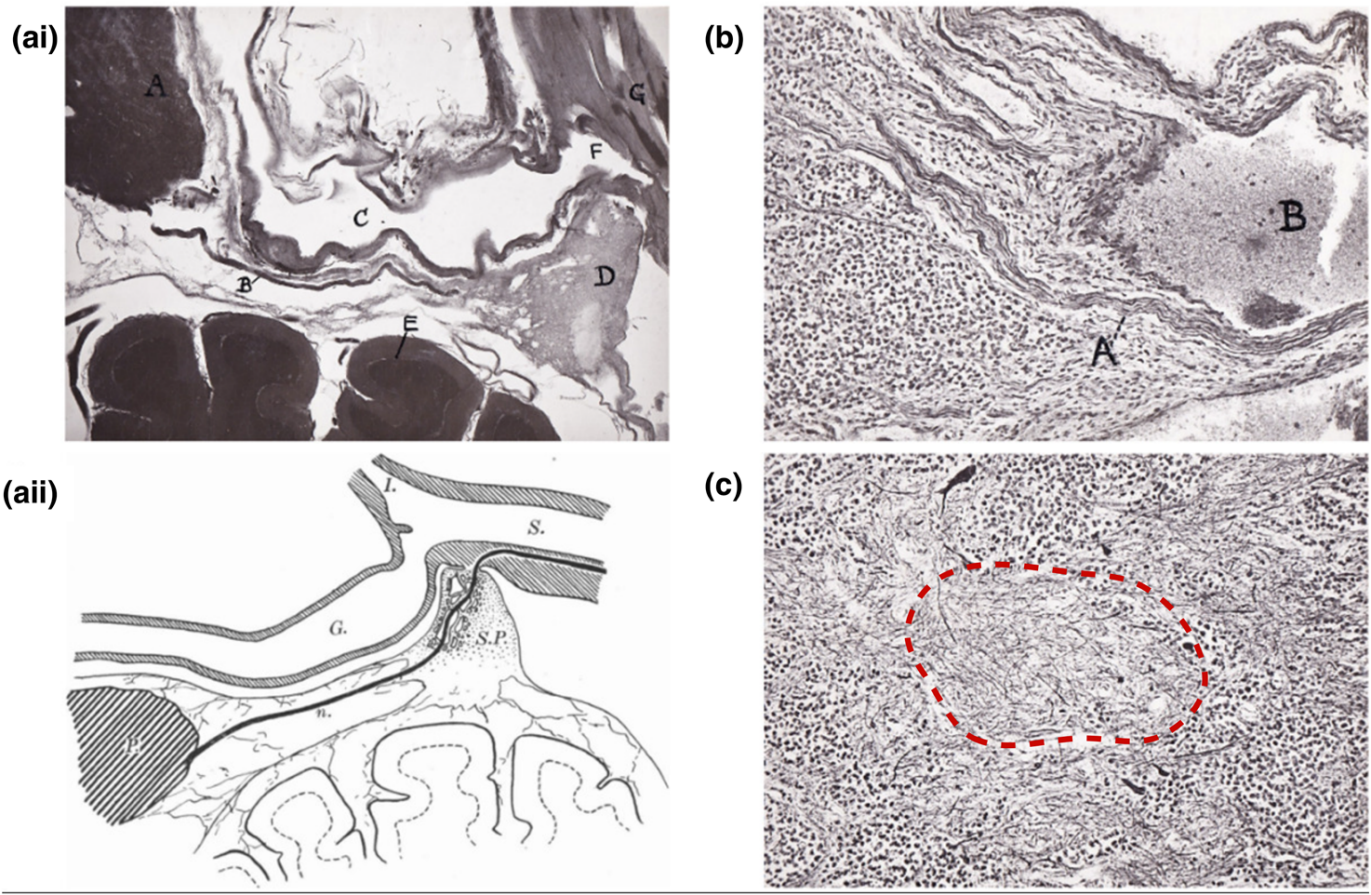
The anatomical relationship between the pineal gland and *nervus conarii*. (ai) Human brain section stained with hematoxylin and eosin (×12). A: Pineal gland. B: *Nervus conarii*. C: Great cerebral vein of Galen. D: Arachnoid granulation. E: Cerebellum. F: Straight sinus. G: Tentorium cerebelli. (aii) Schematic diagram of *nervus conarii* pathway in human brain adapted from Le Gros Clark (1940b). P.: Pineal gland. G.: Great cerebral vein of Galen. S.: Straight sinus. I.: Inferior sagittal sinus. S.P.: Suprapineal arachnoid body. (b) Rhesus macaque brain section stained with protargol (×118) *nervus conarii* coursing along great cerebral vein of Galen. A: *Nervus conarii*. B: Great cerebral vein of Galen. (c) Rhesus macaque brain section through pineal gland (×160) revealing central bundle of nerve fibers (outlined in red) surrounded by conspicuous ganglion cells. Notes from Le Gros Clark: (Relating to Figure ai) “This is a thick section (stained with hematoxylin and eosin) through the pineal gland, and adjacent parts of a human brain. The tip of the pineal gland is seen to the left (A). Issuing from it is a fine neve, the pineal nerve (B), which can be followed for some distance. It runs back in the floor of the great cerebral vein (C) toward a massive arachnoid granulation (D) which projects up from the upper surface of the cerebellum (E). The arachnoid granulation is closely related to the termination of the great cerebral vein where the latter opens into the straight sinus (F) in the tentorium cerebelli (G). The significance and ultimate connexions of the pineal nerve are still quite unknown. The photograph is at a magnification of ×12.” (Relating to Figure b) “Section of the tip of the pineal gland of a monkey, stained with protargol. It shows the ‘pineal nerve’ (A) emerging and passing back in close relationship to the wall of the great cerebral vein (B). The photograph is at a magnification of ×118.” (Relating to Figure c) “Section through the middle of the pineal gland of a monkey, stained with protargol. It shows that in the middle of the gland (at least in the monkey) there is a central core of a dense plexus of fine nerve fibers, scattered in which are some conspicuous ganglion cells. The significance of this nervous tissue is quite unknown. The photograph is at a magnification of ×160.”

Using serial histological sections and silver‐based staining methods (including protargol), Le Gros Clark observed a narrow tract originating within the neuropil of the pineal gland and extending posteriorly along the vein of Galen, eventually reaching the wall of the straight sinus (Figure 6a–c). In one particularly detailed rhesus macaque specimen, he noted a central core of fibers embedded along what he described as “conspicuous ganglion cells” within the gland itself (Figure 6c). He also described a suprapineal arachnoid structure, termed the “suprapineal arachnoid body,” through which the fiber passed before adopting a subendothelial position along the straight sinus. Based on its course and close anatomical relationship with venous structures, Le Gros Clark hypothesized that the nerve played a regulatory role in venous outflow from the pineal region, perhaps acting as part of a ball‐valve‐like mechanism (Figure 6a).

Despite the thoroughness of his anatomical dissections, Le Gros Clark was unable to determine the fiber's destination. He remained agnostic as to whether the *nervus conarii* represented an afferent or efferent pathway, though his functional interpretation implied an efferent role. Interestingly, his findings only partially aligned with earlier observations by Pastori (1928, 1930), who had also traced the fiber and proposed the existence of a “ganglion conarii.” Le Gros Clark did not confirm the presence of such a structure, and this discrepancy, although largely overlooked at the time, would later prove significant.

**FIGURE 7:**
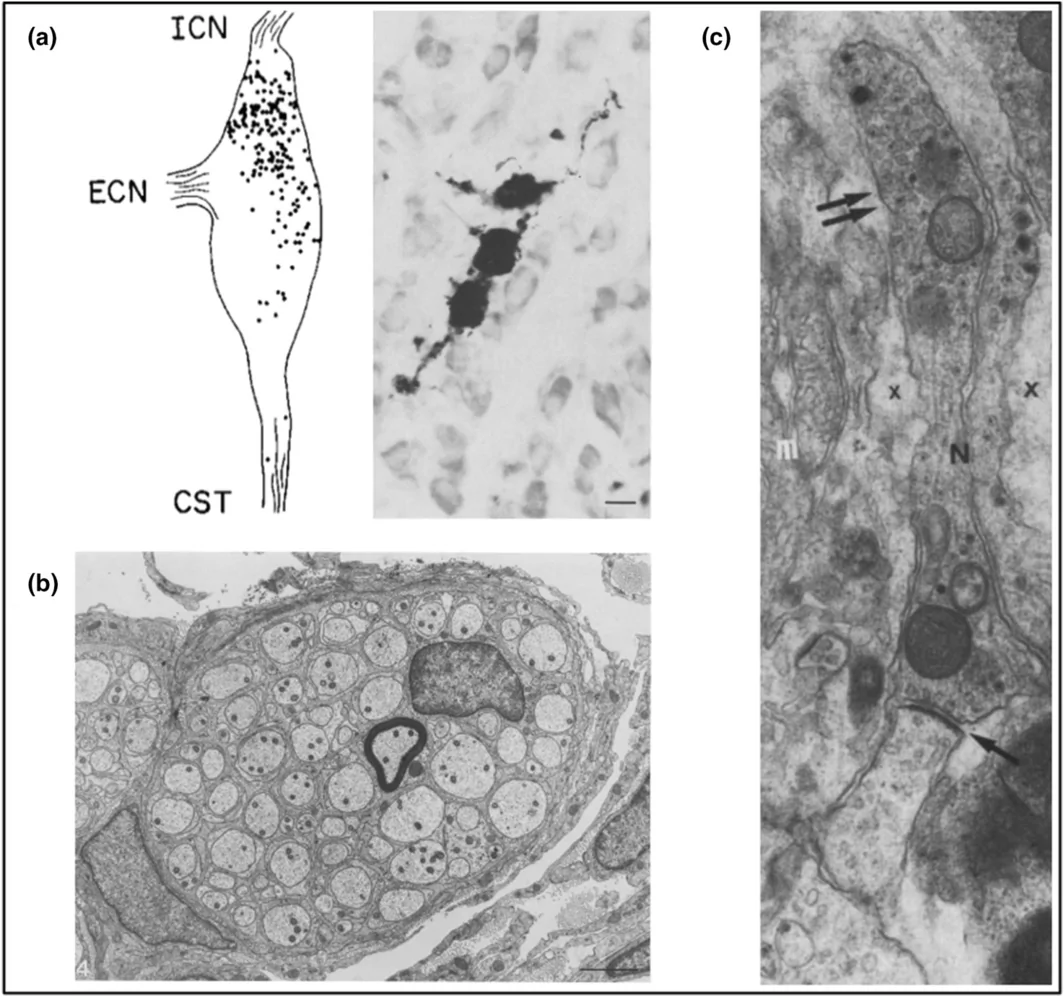
Sympathetic innervation of the pineal gland via the *nervus conarii*. (a) Left: Distribution of neurons in the rat superior cervical ganglion retrogradely labelled with horseradish peroxidase (HRP) following injection of the tracer into the pineal gland. As HRP is transported retrogradely from axon terminals to cell bodies, labelled somata identify neurons projecting to the gland. CST, Cervical sympathetic trunk; ECN, External carotid nerve; ICN, Internal carotid nerve. Right: Three‐HRP‐labelled neurons in the superior cervical ganglion, counterstained with neutral red. Scale bar = 25 μm. Adapted from Bowers et al. (1984). Right: Horseradish peroxidase‐labelled neurons counterstained with neutral red. CST, Cervical sympathetic trunk; ECN, External carotid nerve; ICN, Internal carotid nerve. Scale bar = 25 μm. Adapted from Bowers et al. (1984). (b) Electron micrograph of one of the small nerve bundles comprising the *nervus conarii*, containing predominantly unmyelinated axons with a single myelinated axon. Scale bar = 1 μm. Adapted from Bowers et al. (1984). (c) Electron micrograph of a longitudinally sectioned adrenergic nerve fiber (N) forming a synaptic contact (single arrow) with a presumptive pinealocyte process, with a preterminal varicosity (double arrows) in the same fiber. m, Mitochondria; x, Intraparenchymal extension of the perivascular space. Original magnification ×30,000 as published. A scale bar cannot be derived, as the reduction applied to the original plate is not documented. Adapted from Huang and Lin (1984).

With the development of experimental neuroanatomical techniques in the latter half of the twentieth century, the directionality and function of the *nervus conarii* were definitively clarified. Rodent studies demonstrated that the nerve fibers entering the pineal gland were in fact post‐ganglionic sympathetic axons originating from the superior cervical ganglia. Experimental lesioning of the superior cervical ganglion in rats produced degeneration of these fibers, confirming their afferent nature with respect to the gland (Kappers, 1960). Subsequent tracing studies using horseradish peroxidase retrograde labelling techniques provided more precise maps, in which injection into the pineal gland resulted in dense labelling of ipsilateral superior cervical ganglion neurons, particularly those projecting via the internal carotid nerves (Figure 7a) (Bowers et al., 1984). Ultrastructural analyses reinforced these findings. Electron microscopy revealed that the fibers of the *nervus conarii* terminate in close contact with pinealocytes, often forming varicosities and synaptic‐like junctions (Figure 7b,c) (Huang & Lin, 1984). These terminals release noradrenaline and play a key role in the nocturnal stimulation of melatonin synthesis, a process now central to our understanding of circadian biology. Interruption of this pathway chemically (with 6‐hydroxydopamine sympathectomy) or surgically (with superior cervical ganglionectomy) significantly diminishes melatonin secretion and disrupts the rhythmic firing patterns of pinealocytes (Reuss, 1986; Siaud et al., 1994).

Together, these modern studies have redefined the *nervus conarii* as a paired, post‐ganglionic sympathetic projection from the superior cervical ganglion to the pineal gland, central to circadian regulation. While the true nature of the *nervus conarii* would only be revealed decades later, Le Gros Clark's meticulous anatomical observations, especially along the Galenic venous system, preserved a fragment of physiological mystery that would ultimately underpin one of the most fundamental neuroendocrine rhythms in vertebrate biology, once again underscoring his lasting legacy as a scholar whose curiosity bridged generations of scientific discovery.

### 2.6 The specimen as argument: What distinguishes this collection

Considered together, the specimens presented here share features that are difficult to reproduce with contemporary teaching material. The first is their completeness as clinicopathological units. Each slide is accompanied by a descriptive card in which Le Gros Clark states what is visible in the section and then reconstructs, tract by tract, the deficits the patients would have shown: the loss of postural sense and ataxic gait that follow degeneration of the tract of Goll, or the inability to distinguish one coin from another by touch that follows degeneration of the tract of Burdach. The anatomy is never presented as an end in itself, but as the explanation for a clinical picture, and the card supplies the inferential step between the two. Contemporary atlases and virtual slide libraries reliably supply the image and the label, but less often preserve the reasoning that connects them.

The second is the serial organization of the material. Several cases comprise transverse sections taken at multiple spinal levels from the same patient (Figures 3, 4a,d), so that a degenerating tract can be followed rostrocaudally within a single individual rather than inferred by comparison across unrelated exemplars. Combined with Weigert–Pal staining, which resolves myelinated tracts across the whole section rather than at cellular resolution, this allows the logic of long‐tract anatomy to be read directly from the tissue. The third is irreplaceability. Tabes dorsalis and syringomyelia are now encountered rarely, and the fixed material to teach them cannot be recollected. Multiple sclerosis remains common, but whole‐cord myelin‐stained sections from untreated disease are effectively unobtainable. These specimens document not only a set of diseases but also a state of clinical practice that preceded penicillin, immunomodulation, and neuroimaging, and it is partly this that gives them their continuing pedagogical force.

Digitization has changed how this material is used without displacing the microscope. High‐resolution whole‐slide scanning has made the collection simultaneously accessible, annotatable, and asynchronously available, and has arrested the slow attrition of fading, mounting‐medium degeneration, and breakage to which century‐old preparations are subject. The scanned sections now form part of computer‐assisted neuroanatomy teaching at the University of Oxford (Box 1). What the digital surrogate does not reproduce is the discipline of the microscope itself: focusing through the thickness of a section, navigating a stage to locate a field, and judging staining quality directly. The two are therefore better understood as complementary, the scan extending reach and permanence, the slide preserving the practice. A comparable complementarity holds with respect to modern virtual neuroanatomy resources. Contemporary digital atlases achieve a resolution, spatial continuity, and three‐dimensional reconstruction that no historical collection can approach, but they are overwhelmingly derived from neurologically normal tissue and oriented toward reference mapping of normal architecture. Annotated human pathological material at whole‐section scale remains comparatively scarce in openly available digital collections, and it is here that Le Gros Clark's slides retain their value: not as a lower resolution substitute for contemporary resources, but as a record of disease processes that those resources do not represent and, in several cases, could no longer be assembled to represent.

## 3 CONCLUSION

The preserved neuroanatomical slides of Sir Wilfrid Edward Le Gros Clark represent more than historical artifacts. They are a testament to a pedagogical philosophy that fused scientific rigor with humanistic curiosity. Through these materials, Le Gros Clark invited students not merely to memorzse, but to observe, interpret, and understand the functional significance of anatomical structures in health and disease. His teaching style, informed by clinical context and comparative anatomy, redefined anatomical education at the University of Oxford and beyond. The clinical cases are so didactic and clear that they are still used in the training of second‐year medical students (Chang & Molnár, 2015).

His legacy is etched not only in his writings and students, but physically in the fabric of the University itself. The Le Gros Clark building housed the Department of Human Anatomy for over a century and bore witness to successive generations of anatomical discovery. Today, the annual Sir Wilfrid Le Gros Clark Prize Lecture Series (https://www.dpag.ox.ac.uk/named‐lectures/wilfrid‐le‐gros‐clark‐lecture‐series) continues to honor his intellectual spirit, convening experts who build on the questions he first posed. That many of his slides remain in use, or in active scholarly reinterpretation, underscores the enduring relevance of his work.

In revisiting these teaching specimens, we are reminded that anatomy is not merely the study of static structures, but a living dialogue between past and present, between pedagogy and progress. Le Gros Clark's legacy challenges us to teach anatomy not as a closed canon, but as an evolving narrative anchored in observation, enriched by history, and propelled by inquiry.

## AUTHOR CONTRIBUTIONS

SAN: Conceptualization, data analysis, and manuscript writing and review. KMK: Conceptualization, data analysis, and manuscript writing and review. KT: Conceptualization, data analysis, and manuscript writing and review. MA: Conceptualization, data analysis, and manuscript writing and review. GG: Conceptualization, data analysis, and manuscript writing and review. RW: Conceptualization, data analysis, and manuscript writing and review. ZM: Conceptualization, data analysis, data acquisition, manuscript writing and review, obtaining funding and supervision.

## FUNDING INFORMATION

The Oxford History of Medical Sciences Project was supported by The Wellcome Trust and Federation of European Neuroscience Societies History of Science Grants. ZM's laboratory was supported by the following funding sources: BBSRC Project Grant [BB/X008711/1], MRC Project Grant [MR/W029073/1], Einstein Stiftung Berlin with Prof Britta Eickholt at Charité‐Universitätsmedizin Berlin, Germany, as part of ZM being Einstein Fellow at Charité Universitätsmedizin Berlin, Oxford Martin School Grant (repairing the brain with 3D‐printed neural tissues) (Bayley, Szele, Molnár), and St John's College Research Centre Grant. SAN is supported by a private donation from the Morris family to better understand the pathogenesis of lissencephaly. MA and GG are supported by NIH‐OxCam graduate studentships. This research was supported in part by the Intramural Research Program of the National Institutes of Health (NIH). The contributions of the NIH authors were made as part of their official duties as NIH federal employees, are in compliance with agency policy requirements, and are considered Works of the United States Government. However, the findings and conclusions presented in this paper are those of the authors and do not necessarily reflect the views of the NIH or the U.S. Department of Health and Human Services.

## CONFLICT OF INTEREST STATEMENT

The authors have no relevant conflicts of interest to declare.

## Data Availability

The digitized collection of Le Gros Clark's teaching material is available at DOI: 10.25446/oxford.33257403.
